# Supplementary material for: Deletion of wfs1 Impairs Oligodendrocyte Precursor Cells Dorsal Distribution and Myelination Through the wfs1-hmgcs1 Axis in Zebrafish
Source: Biology (Basel). 2026 Aug 16;15(16):1402. doi: 10.3390/biology15161402 (PMC13510151; doi:10.3390/biology15161402)
Supplement: Supplementary file 1 [file biology-15-01402-s001.zip › File S1-Transcriptome sequencing data and differentially expressed genes (DEGs) in wfs1-- zebrafish at 5 dpf/2.KEGG/wfs1KOvsWT/ALL/src/dre00030.html]

dre00030


- dre:560827

- Up regulated genes

560827\_1(4.8362689625617)

- dre:407673

- Down regulated genes

407673(-1.5435670751036)

- dre:568001

- Up regulated genes

568001\_3(1.40501062189015)

- Down regulated genes

568001\_2(-2.92834331323733)
- dre:561416

- Up regulated genes

561416\_1(3.15253579212291)

- Down regulated genes

561416(-4.80431336765849)

- dre:406762

- Up regulated genes

406762\_1(10.4018062903395)

- dre:570579

- Up regulated genes

570579\_1(5.01448411924817)

- dre:403070

- Up regulated genes

403070\_1(2.42915861580394)

- Down regulated genes

403070(-1.45615173353575)

- dre:406762

- Up regulated genes

406762\_1(10.4018062903395)

- dre:570579

- Up regulated genes

570579\_1(5.01448411924817)

Close
